# *KRAS* mutation analysis of washing fluid from endoscopic ultrasound-guided fine needle aspiration improves cytologic diagnosis of pancreatic ductal adenocarcinoma

**DOI:** 10.18632/oncotarget.13864

**Published:** 2016-12-10

**Authors:** Kyung Joo Park, Jung Yoon Lee, Kyun Jong Lee, Taek Kyu Lee, Yoon-La Choi, Hyuck Kwang Lee

**Affiliations:** ^1^ Division of Gastroenterology, Department of Medicine, Samsung Medical Center, Sungkyunkwan University School of Medicine, Seoul, Korea; ^2^ Department of Pathology, Samsung Medical Center, Sungkyunkwan University School of Medicine, Seoul, Korea; ^3^ Department of Health Sciences and Technology, Samsung Advanced Institute for Health Sciences & Technology, Sungkyunkwan University, Seoul, South Korea

**Keywords:** KRAS gene mutation, endoscopic ultrasound-guided fine needle aspiration, pancreatic ductal adenocarcinoma

## Abstract

EUS-FNA becomes one of the most important diagnostic modalities for PDACs. However, acquired tissue specimens were sometimes insufficient to make a definite cytological diagnosis. On the other hand, *KRAS* mutation is the most frequently acquired genetic alteration found more than 90% of PDACs. To investigate the way to improve diagnostic accuracy for PDACs using both cytological examination and KRAS mutation analysis would be a great help. Therefore, the aims of this study were to evaluate usefulness of conventional cytological examination combined with *KRAS* mutation analysis with modified PCR technology to improve the sensitivity and the accuracy. We enrolled 43 patients with solid pancreatic masses and 86 EUS-FNA specimens were obtained. During the EUS-FNA, the needle catheter was flushed with 2 cc of saline and the washed fluid was collected for *KRAS* mutation analysis for the first 2 passes; PNAClamp™ *KRAS* Mutation Detection Kit. There were 46 specimens from the 23 PDACs and 40 specimens from the 20 other pancreatic diseases. The sensitivity, specificity and accuracy were as follows; conventional cytopathologic examination: 63%, 100% and 80%; combination of cytopathologic examination and K-ras mutation analysis: 87%, 100% and 93%. Furthermore, KRAS mutation was detected 11 out of 17 PDAC samples whose cytopathology results were inconclusive. *KRAS* mutation analysis with PNAClamp™ technique using washing fluid from EUS-FNA along with cytological examination may not only improve the diagnostic accuracy of PDACs, but also establish the platform using genetic analysis which would be helpful as diagnostic modality for PDACs.

## INTRODUCTION

The prognosis for pancreatic ductal adenocarcinoma (PDAC) remains dismal with 5-year survival rates of 6% and 85–90% of PDACs are found out to be inoperable at the time of diagnosis [1]. One of the major limitations to study tumor biology of PDACs was due to difficulties acquiring tissue specimens from most of unresectable PDACs. The majority of what is known about PDACs so far comes from the surgical specimens which only comprise 20% of the PDAC population. The remaining 80% of patients have locally advanced or metastatic disease in these patients little to no data is available because of access to tissues. Recently, endoscopic ultrasound-guided fine needle aspiration (EUS-FNA) method has become a gold standard in the diagnosis of PDACs and became the most promising tool as a diagnostic modality [2–5]. However, the aspirated specimen obtained by this technique sometimes insufficient to make a definite cytopathological diagnosis [3, 6–9]. Therefore, it is very important to investigate the diagnostic modality with high accuracy and being capable of getting sufficient tissue for PDACs.

The *KRAS* gene is the locus for the *c-k-ras* proto oncogene located on the short arm of chromosome 12 (12q) and the majority of mutations have been found at *KRAS* codons 12 and 13. *KRAS* mutations are the most frequently acquired genetic alteration in PDAC, and detecting this mutation from pancreatic tissue has been helpful in the diagnosis of pancreatic cancer [5, 10]. Previous studies revealed that the combined modality of cytopathology and *KRAS* mutations detection could improve the sensitivity of the diagnosis of pancreatic cancer [5, 10, 11]. Although conventional PCR amplification followed by direct sequencing has been the gold standard for the detection of *KRAS* gene mutation to date, this technique has low sensitivity [12, 13]. It can detect only mutant sequences that contain more than 25% of total gene content [13, 14]. To overcome this drawback, several alternative modalities were devised. The peptide nucleic acid (PNA)-mediated PCR clamping technique is a modified PCR technology by using optimized PNA probes [12]. PNA is a synthetic DNA analog in which the phosphodiester backbone is replaced by a peptide-like repeat of the (2-aminoethyl)-glycine chain [15, 16]. The above mentioned structural characteristics lead to the thermal stability of PNA-DNA duplexes than the corresponding DNA-DNA duplexes [17]. The PNA has tight binding to the wild type DNA fragments and eventually resulted in no amplification for wild type DNA [17]. On the other hands, the PCR amplification is processed for multiplication In the case of mutated genes, such as SNPs, of the mutated DNA sequences [17]. This technique can detect *KRAS* gene mutation using a tiny amount of specimen obtained by EUS-FNA [18–20]. The combination of conventional cytopathological diagnosis and *KRAS* mutation analysis with modified PCR technology was investigated to improve the sensitivity of diagnosis for the PDAC [5].

## RESULTS

### Baseline characteristics

The total of 43 study patients underwent EUS-FNA and tissue specimen was acquired successfully (Table 1). Of the 43 patients there were a total of 26 (60.5%) males and 17 (39.5%) females. The mean age in the group was 61.4±13.3 years, range 19-85 years. The locations of the lesions in pancreas were as follows; head and uncinate of the pancreas in 24 patients, body in 12 patients and tail in 7 patients. The average size of the mass in study patients was 33.0±12.3 mm. There was no significant difference in size among the pancreas mass according to the final diagnosis; PDAC (median 31.8 mm, 10 ~ 80), pancreas neuroendocrine tumor (PNETs; median 25.2 mm, range 16 to 40), and inflammatory mass due to pancreatitis (27.5 mm, range 20 to 30). EUS-FNA was performed with 22 gauge or 25 gauge needle and the average number of needle pass was 3.5 times (Table 1). Among the 43 patients, final diagnoses were as follows; 23 PDACs and 20 non-PDACs: PNET, inflammatory mass with pancreatitis, metastatic GB cancer, solid pseudopapillary neoplasm (SPN), serous cystadenoma and metastatic renal cell carcinoma (RCC) (Table 2).

**Table 1:** Baseline characteristics (n=43)

Sex (male/female)	26/17
Age (y)	61.4±13.3
Location (head & uncinate/body/tail)	24/12/7
Size (mm)	33.0±12.3
Needle (22G:25G)	32:11
Passing number (times)	3.5 ±0.8

**Table 2:** Final diagnosis (n=43)

PDACs*	23
Non-pancreatic cancer	20
Neuroendocrine tumor	5
Pancreatitis	4
Metastatic GB cancer	2
SPN+	2
Serous cystadenoma	2
Metastatic RCC	1
Other benign disease	4
*PDAC: pancreatic ductal adenocarcinoma, +SPN: Solid Pseudopapillary Neoplasm.	

### Cytopathologic diagnosis

**Figure 1:**
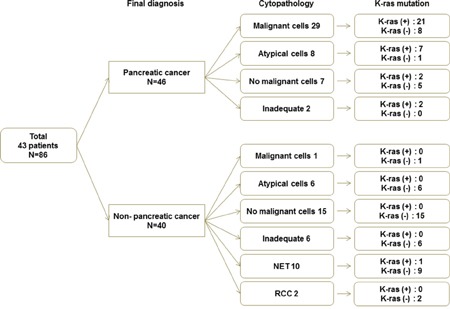
Final diagnosis, cytopathology and KRAS mutation

This study was to investigate the diagnostic modality which would increase the accuracy and sensitivity in evaluating minimal specimens from EUS-FNA. In addition, EUS-FNA for pancreatic mass usually have not sufficient amount of tissue and therefore, to develop diagnostic technique which is capable of adding more information in diagnosis of PDAC would be very useful in clinical practice. There were 86 study samples and the final diagnosis were the followings; 46 PDACs and 40 non-PDACs such as 5 PNETs, 4 pancreatitis, 2 metastatic GB cancers, 2 SPNs, 2 serous cystadenomas, 1 metastatic RCC and 4 other benign diseases (Table 2, Figure 1). Among the 46 PDAC samples, 29 samples were positive for adenocarcinoma cells in cytology samples. There were 17 samples with negative results from the cyotologic examination such as; atypical cells, inadequate samples due to low cellularity and without malignant cells. The sensitivity, specificity and overall accuracy for cytopathologic diagnosis of pancreatic cancer were 63%, 100%, and 80%, respectively (Table 3).

Table 3

**Table:** (A) Cytopathological examinations

	Pathology Y	Pathology N
PDAC Y (N=46)	29	17
PDAC N (N=40)	0	40

**Table:** (B) Combined cytopathological examinations and K-ras mutation analysis

	Pathology/K-ras Y	Pathology/K-ras N
PDAC Y	40	6
PDAC N	0	40

**Table:** (C) Sensitivity, specificity and overall accuracy of the diagnosis of PDACs

	Sensitivity (%)	Specificity (%)	Accuracy (%)
Cytopathology	29/46 (63%)	40/40 (100%)	68/86 (80%)
Combination of both	40/46 (87%)	40/40 (100%)	80/86 (93%)

### Detection of *KRAS* mutations

Among the 46 PDAC samples, 29 samples were positive for adenocarcinoma cells in cytology samples. There were 17 samples with negative results from the cyotologic examination such as; atypical cells, inadequate samples due to low cellularity and without malignant cells. K-ras mutation analysis from the washing fluid of cytology negative samples was as follows; 11 out of 17 samples were positive for K-ras mutation analysis. Also, there were also 40 non–PDAC samples and 39 samples were found no K-ras mutation except for one sample whose histologic diagnosis from EUS-FNA was PNET (Figure 1).

### Combination of cytopathology and *KRAS* mutation analysis

**Figure 2:**
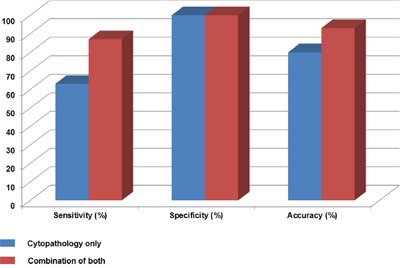
Sensitivity, specificity and accuracy for the diagnosis of PDAC

Tissue specimens from the EUS-guided FNA & FNB were acquired and after the procedure, the needles and apparatus would be disposed. However, we have used washing fluid passing through the needles and analyzed K-ras mutation status. Using washing fluid samples was our original idea in this study and it usually wouldn't be existed in routine clinical practice. There were 86 study samples and the final diagnoses were the followings; 46 PDACs and 40 non-PDACs: 5 PNETs, 4 pancreatitis, 2 metastatic GB cancers, 2 SPNs, 2 serous cystadenomas, 1 metastatic RCC and 4 other benign diseases. Among the 46 PDAC samples, the 29 samples were positive and rest of the 17 samples were negative or not definitive for ductal adenocarcinoma cells from the cytopathologic examination. However, *KRAS* mutant gene was detected in 11 out of 17 samples and it contributed to the diagnosis of PDACs. Also, there were 39 out of 40 non–PDAC samples found no K-ras mutation except for one sample whose histologic diagnosis from EUS-FNA was pancreas neuroendocrine tumor. In Table 3, each cytopatholgical examination and combined result was as follows. In cytopathological examinations, sensitivity, specificity and accuracy were 63%, 100% and 80% (Figure 2). When it is combined with K-ras mutation analysis from washing fluid during EUS-FNA & FNB, they became 87%, 100% and 93% respectively (Table 3, Figure 2). As we have described in the above paragraphs, combination of cytopathology and KRAS mutation analysis was very helpful as diagnostic modality for PDACs. There was a 45-year old woman presented with epigastric and back pain among study patients. Abdominal CT scanning showed a solid mass like lesion with cystic changes in the head of pancreas accompanied by bile duct dilatations. FNA was performed and two separate aspirated samples were obtained. Cytopathological diagnosis for the first EUS-FNA specimen was “consistent with adenocarcinoma” and “a few inflammatory cells present” for the second one. In spite of the inconsistency of cytopathological examinations, *KRAS* mutations were detected in both of samples.

### Complications

There were no complications associated with EUS-FNA in study patients.

## DISCUSSION

PDAC has one of the lowest survival rate among the malignant solid tumors and it is approximately 5% for all stages combined [1]. Majority of PDACs usually are found in locally advanced or metastatic disease at the time of diagnosis. Early detection of PDAC and complete surgical resection is the only chance to improve 5-year survival rate up to 20%. Recently EUS-FNA has been established as a gold standard for the diagnosis of PDAC [2–5]. EUS-FNA is more safe and effective than percutaneous ultrasonography- or CT-guided FNA, because it can avoid vessels by using color doppler and make shorter track to the intrapancreatic masses. However, EUS-FNA technique still implies some drawbacks like the relatively low negative predictive value (NPV) for the diagnosis of PDACs [21, 22]. It is noteworthy to point out that a negative result of cytopathology cannot rule out pancreatic cancer, because the accuracy of cytopathologic diagnosis from FNA specimens depends upon accurate sampling [3]. Moreover, even if we got the specimen from the accurate target, the amount of aspirated material could be too small to make a definite cytopathologic diagnosis and additional punctures could be needed. To overcome these drawbacks, a number of different diagnostic modalities have been investigated.

*KRAS* mutation is one of the most frequent and important genetic alterations in PDAC, and the combined modality of cytopathology and *KRAS* mutation analysis could improve the sensitivity in diagnosing PDACs [5, 10, 11]. Takahashi et al. analyzed *KRAS* mutation in EUS-FNA specimens by direct sequencing to facilitate a differential diagnosis between PDAC and focal pancreatitis [10]. They reported the sensitivity according the following diagnostic modalities; cytopathology, *KRAS* mutation analysis and the combination of cytopathology and *KRAS* mutation analysis were 84%, 74% and 94%, respectively [23]. As above study showed, the sensitivity for the diagnosis of PDAC improved markedly, from 82% to 94%, in cases of PDAC when the presence of the *KRAS* mutation was taken into consideration [10]. But more punctures of EUS-FNA should be performed to carry out *KRAS* mutation analysis [10]. Tada et al. performed the quantitative analysis of *KRAS* mutation by using washing fluid from FNA needle catheter [5]. In their study, the sensitivity of cytopathology, *KRAS* mutation analysis and the combination of cytopathology and *KRAS* mutation analysis was 62%, 77% and 81%, respectively [23]. They also reported that no *KRAS* mutations had been observed in non-cancer cases. *KRAS* mutation analysis supplemented conventional cytopathology of EUS-FNA, up by 19%. But the detection rate of *KRAS* mutation by quantitative analysis technique is slightly lower compare to other studies used resected pancreatic specimens [5, 10]. The conventional PCR amplification technique followed by direct sequencing can detect only mutant sequences that contain > 25% of total gene content [14].

To improve the sensitivity of pancreatic cancer diagnosis and to avoid additional punctures, we have applied the *KRAS* mutation analysis technique with modified PCR technology using the washing fluid after EUS-FNA. The PNA-mediated PCR clamping technique is a modified PCR technology by using optimized PNA probes [12]. PNA is a synthetic DNA analog, phosphodiester backbone is replaced by a peptide-like repeat of the (2-aminoethyl)-glycine chain [15, 16]. The structural characteristics lead to the thermal stability of PNA-DNA duplexes over the corresponding DNA-DNA duplexes [17]. The tight binding of PNA to the wild type DNA fragments results in no amplification of the wild type DNA fragments. However, in the case of mutated genes, especially with SNPs, the PCR amplification is processed for multiplication of the mutated DNA sequences. This technique can detect *KRAS* gene mutation using a tiny amount of specimen obtained by EUS-FNA [18–20].

Jeoung et al. evaluate the efficacy of PNA-clamp real-time PCR technique compared to the conventional direct sequencing [13]. They reported that PNA-based real-time PCR clamping technique was more sensitive than direct sequencing for detecting *KRAS* mutation [13, 24]. The PNA-based real-time PCR clamping detected 1% of mutant genes in 1 ng DNA. Kobunai et al. reported a sensitivity of 0.4% in 2 ng DNA using cell lines and the PNA-clamp real-time PCR SYBR assay [25].

In this study, we performed the PNA mediated PCR clamping technique using washing fluid after EUS-FNA and the combination of cytopathology and *KRAS* mutation analysis showed a sensitivity of 87.0%, up by 26.1% compared with cytopathologic examination alone. It is noteworthy that this method detects *KRAS* mutation without additional invasive try or efforts, because it can be performed only with the washing fluid of EUS-FNA needle after puncture.

Definitely, *KRAS* mutation analysis could be a supplement to the conventional cytopathological diagnosis, when only “atypical cells or suspicious of malignancy” was reported in spite of clinically strong suspicion of malignancy or when aspirated specimens were too small to make a definite diagnosis. Cytopathologic examination is a gold standard diagnostic modality and we had the sensitivity and accuracy of 63% and 80% respectively. Compared to the previous studies which reported 62~94% of sensitivity, our result show only 63% of low sensitivity [5, 10, 26]. We have included the 4 samples with the inadequate specimens for cytopathological examination, but *KRAS* mutation was detected for all those samples. Therefore, For example, one case was 54-year old man who presented with epigastric discomfort. Abdomen CT scanning showed about 5.3 cm sized mass lesion in the pancreatic tail. Four times to and fro motions were done and four separate samples were obtained. Cytopathological examinations for the first two specimens were reported as ‘a few atypical cell clusters’ and ‘suspicious for malignancy’, but ‘Scanty cellular smear’ for the last two samples. In spite of the uncertainty of cytopathological diagnosis, *KRAS* mutation was detected. The final diagnosis was PDAC after the surgical confirmation.

On the other hands, there were 39 out of 40 non–PDAC samples without K-ras mutations except for one sample whose histologic diagnosis was PNETs. There is a possibility that K-ras mutation was picked up from the adjacent PanIN lesions. However, this study was performed by endoscopic experts who had performed EUS-FNA more than 2,000 cases at the time of study enrollment and it is very hard to miss the target lesions. Moreover, K-ras mutation in PNET has been reported in several studies. Jiao et al first reported genomic landscape of pancreas neuroendocrine tumors [27]. They had 10 PNETs for the discovery set and found 8 to 23 mutations per tumor, however, the frequently mutated genes in PDACs such as TP53, KRAS, CDKN2A and SMAD4 were not found in their PNET samples. Interestingly, Jiao et al studied 43 PNETs in Chinese population and found out the frequently mutated genes as follows; DAXX/ATRX, KRAS, MEN1, mTOR pathway genes (PTEN, TSC2), SMAD4/DPC, TP53 and VHL were mutated in 54%, 11%, 54%, 3%, 14% and 41% respectively [27]. The mutation rates of above mentioned genes in Chinese PNET patients are different from those in Caucasians that there were a higher number of mutated genes [27]. Furthermore, the DAXX/ATRX and KRAS gene mutations are correlated with a poor prognosis of patients with PNETs [27]. We don't know yet whether those different results are due to the ethnic differences or picking up adjacent PanIn lesion either. False positive for K-ras mutation issue needs to be further investigated.

Most of PDACs are unresectable and there are often not sufficient tissue samples which can be used for diagnosis and not to mention research material of PDAC biology. We have tried to increase the diagnostic accuracy without using additional tissue specimens. Here, we report both combined modalities cytopathological examination and KRAS mutation analysis with PNA mediated PCR clamping technique (PNAClamp™) from EUS-FNA have successfully demonstrated its usefulness as diagnostic modality for PDACs. Moreover, it can be performed without additional efforts since it uses only washing fluid after EUS-FNA procedure. Furthermore, KRAS mutation was detected 11 out of 17 PDAC samples whose cytopathology results were inconclusive. Therefore, *KRAS* mutation analysis with PNAClamp™ technique using washing fluid from EUS-FNA along with cytopathological examination may not only improve the diagnostic accuracy of PDACs, but also establish the platform of genetic analysis which would be helpful as diagnostic modality for PDACs.

## MATERIALS AND METHODS

### Patients

A total of 43 consecutive patients were prospectively enrolled in this study and underwent EUS-FNA & FNB procedures at Samsung Medical Center, the tertiary teaching hospital. The value of *KRAS* mutational analysis from the washing fluid during EUS-FNA samples was evaluated whether it can be helpful along with the conventional cytopathology. The inclusion criteria was as follows; the patients who were found to have pancreatic mass on CT scan or MRI and those scheduled to undergo EUS-FNA for further evaluation. The patients with pancreatic mass and definite evidence of metastasis were excluded in this study. Informed consent for EUS-FNA was obtained from all patients, and this work was performed in accordance with the humane and ethical principles of research set forth in the Helsinki guidelines.

### EUS-FNA technique

Patients received pharyngeal local anesthesia and were sedated with injections of 2 to 12 mg (average 4.8 mg) of midazolam. Standard EUS was first performed using a radial scanning echoendoscope (JF UM20; Olympus, Tokyo, Japan) and a 22-gauge or 25-gauge needle (NA-10J-1 or NA-11J-KB; Olympus, Tokyo, Japan) to identify and evaluate the lesions. EUS-guided FNA was then performed using a curved linear array echoendoscope (FG-36 UA; Pentax/Hitachi, Tokyo, Japan) with a GIP/Medi-Globe 22-gauge, 10-cm needle (GIP Medizin Technik, Grassau, Germany). After scanning the pancreatic masses with pulse and color Doppler methods to evaluate the lesion and presence of adjacent vessels, a catheter was inserted into the biopsy channel and the needle tip was advanced incrementally under real-time endoscopic guidance into the lesion. The EUS-FNAs were either transgastric or transduodenal, depending on the site of the lesion. The stylet was removed and suction was applied through a 20-ml syringe as the needle moved back and forth two to six times (average 3.5 times) within the lesion. The needle was then retracted into the catheter and the entire catheter was removed. The aspirated materials were placed onto glass slides by releasing the syringe and direct smears were prepared. FNA samples were stained using the rapid Hematoxylin and Eosin (H&E) staining technique. Then the needle catheter was flushed with 2 ml of saline and residual materials were collected for DNA extraction. Those collected samples were frozen right after EUS-guided FNA and the storage kept the specimens in −80°C.

### Cytopathological diagnosis

Definitive cytopathological diagnosis was stated by a single pathologist, blinded to the result of EUS, after staining. The pathologist was provided a clinical history as well as the site and size of the mass lesion. For classification, the cytology specimens were interpreted as benign, atypical or suspicious of malignancy, malignant, or inadequate specimens if representative material was not present. The only judgment of “malignant” was accepted as a definite diagnosis of malignancy.

### Analysis of *KRAS* gene mutation

**Figure 3:**
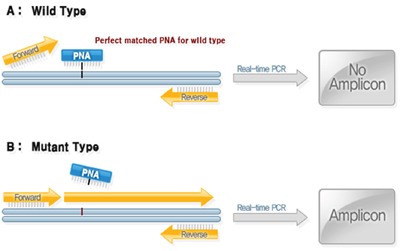
Analysis of KRAS gene Mutation: PNAClamp KRAS Mutation Detection Kit

DNA was extracted from aspirated specimens by high pure PCR template preparation kit (Roche, Mannheim, Germany), according to the manufacturer's instructions, and the amount of DNA was measured by Nano Drop Product (Thermo Scientific, Wilmington, DE). The DNAs were diluted to a concentration representing 50 ng/μL for the test. *KRAS* gene was analyzed by PNAClamp™ KRAS Mutation Detection Kit (Panagene, Daejeon, Korea). This assay is based on PNA-mediated real-time PCR clamping technology. PNA is a synthetic DNA analog in which the phosphodiester backbone is replaced by a peptide-like repeat formed by (2-aminoethyl)-glycine units (Figure 3). Since PNA contains no charged phosphate groups, the binding between PNA/DNA is stronger than between DNA/DNA due to the lack of electrostatic repulsion (Figure 3). Briefly, PCR amplification was performed in a total volume of 20 μL that contained 50 ng of DNA, 13 μL of real-time SYBR Green PCR master mix and each of the primers and PNA probes for codons 12/13, respectively. The PCR control lacked a PNA probe. The PCR cycling conditions were at 94°C for 5 minutes followed by 40 cycles of 94°C for 30 sec, 70°C for 20 sec, 63°C for 30 sec, and 72°C for 30 sec and a final extension of 72°C for 5 minutes. The PNA probe was designed to hybridize completely to the wild-type *KRAS* allele to suppress amplification of wild-type target, thereby enhancing preferential amplification of mutant sequences by competitively inhibiting DNA primer binding to wild-type DNA. PCR efficiency was determined by measuring the threshold cycle (Ct) value. Obtain Ct values for the control and mutation assays by observing the SYBRGreen amplification plots. Delta-Ct values were calculated as the Ct value of the PCR with the PNA control minus the Ct value of the PCR of the samples. The higher delta-Ct value means that the mutant was efficiently amplified. The cutoff value of 2.0 was used for determining the presence of mutant DNA. Theses molecular analyses were provided by Panagene (Daejeon, Korea) in exchange for studying a large number of clinical samples for the diagnosis of pancreatic cancer. Permission to disclose molecular results without notification of the patient's clinical information was obtained in advance from the company.

### Statistical analysis

Statistical differences between the 2-paired results obtained from cytopathologic diagnosis and the combination of conventional cytopathology and *KRAS* mutation analysis were analyzed by the McNemar test. The t-test was used for analysis of tumor size. SPSS Statistical Analysis Software (v19; SPSS, Chicago, IL, USA) was used for statistical analysis. A *p*-value of <0.05 was considered statistically significant.

### Abbreviations

PDAC; pancreatic ductal adenocarcinoma, EUS-FNA; endoscopic ultrasound fine needle aspiration, EUS-FNB; endoscopic ultrasound fine needle biopsy, PNET; pancreas neuroendocrine tumor, SPN; solid pseudopapillary neoplasm, RCC; renal cell carcinoma.

LJK and LKT designed the study and discussed with data.

LKH designed the study, provided study samples, performed a paper work for approval of ethical committee, analyzed the data and drafted the manuscript.

All authors read and approved the final manuscript

## References

[1] Warshaw AL, Fernandez-del Castillo C (1992). Pancreatic carcinoma. The New England journal of medicine.

[2] Wiersema MJ, Vilmann P, Giovannini M, Chang KJ, Wiersema LM (1997). Endosonography-guided fine-needle aspiration biopsy: diagnostic accuracy and complication assessment. Gastroenterology.

[3] Chang KJ, Nguyen P, Erickson RA, Durbin TE, Katz KD (1997). The clinical utility of endoscopic ultrasound-guided fine-needle aspiration in the diagnosis and staging of pancreatic carcinoma. Gastrointest Endosc.

[4] Bhutani MS, Hawes RH, Baron PL, Sanders-Cliette A, van Velse A, Osborne JF, Hoffman BJ (1997). Endoscopic ultrasound guided fine needle aspiration of malignant pancreatic lesions. Endoscopy.

[5] Tada M, Komatsu Y, Kawabe T, Sasahira N, Isayama H, Toda N, Shiratori Y, Omata M (2002). Quantitative analysis of K-ras gene mutation in pancreatic tissue obtained by endoscopic ultrasonography-guided fine needle aspiration: clinical utility for diagnosis of pancreatic tumor. Am J Gastroenterol.

[6] Binmoeller KF, Thul R, Rathod V, Henke P, Brand B, Jabusch HC, Soehendra N (1998). Endoscopic ultrasound-guided, 18-gauge, fine needle aspiration biopsy of the pancreas using a 2.8 mm channel convex array echoendoscope. Gastrointest Endosc.

[7] Harada N, Kouzu T, Arima M, Isono K (1996). Endoscopic ultrasound-guided histologic needle biopsy: preliminary results using a newly developed endoscopic ultrasound transducer. Gastrointest Endosc.

[8] Wiersema MJ (2001). Accuracy of endoscopic ultrasound in diagnosing and staging pancreatic carcinoma. Pancreatology.

[9] Yamao K, Sawaki A, Mizuno N, Shimizu Y, Yatabe Y, Koshikawa T (2005). Endoscopic ultrasound-guided fine-needle aspiration biopsy (EUS-FNAB): past, present, and future. Journal of gastroenterology.

[10] Takahashi K, Yamao K, Okubo K, Sawaki A, Mizuno N, Ashida R, Koshikawa T, Ueyama Y, Kasugai K, Hase S, Kakumu S (2005). Differential diagnosis of pancreatic cancer and focal pancreatitis by using EUS-guided FNA. Gastrointest Endosc.

[11] Salek C, Benesova L, Zavoral M, Nosek V, Kasperova L, Ryska M, Strnad R, Traboulsi E, Minarik M (2007). Evaluation of clinical relevance of examining K-ras, p16 and p53 mutations along with allelic losses at 9p and 18q in EUS-guided fine needle aspiration samples of patients with chronic pancreatitis and pancreatic cancer. World J Gastroenterol.

[12] Kwon MJ, Lee SE, Kang SY, Choi YL (2011). Frequency of KRAS, BRAF, and PIK3CA mutations in advanced colorectal cancers: Comparison of peptide nucleic acid-mediated PCR clamping and direct sequencing in formalin-fixed, paraffin-embedded tissue. Pathol Res Pract.

[13] Jeong D, Jeong Y, Lee S, Lee H, Lee W, Kim H, Park D, Park S, Mu W, Cho HD, Oh MH, Lee SS, Yang SH, Kim CJ (2012). Detection of BRAF (V600E) Mutations in Papillary Thyroid Carcinomas by Peptide Nucleic Acid Clamp Real-Time PCR: A Comparison with Direct Sequencing. Korean journal of pathology.

[14] Pao W, Ladanyi M (2007). Epidermal growth factor receptor mutation testing in lung cancer: searching for the ideal method. Clin Cancer Res.

[15] Nielsen PE, Egholm M, Berg RH, Buchardt O (1991). Sequence-selective recognition of DNA by strand displacement with a thymine-substituted polyamide. Science.

[16] Miyake M, Sugano K, Kawashima K, Ichikawa H, Hirabayashi K, Kodama T, Fujimoto H, Kakizoe T, Kanai Y, Fujimoto K, Hirao Y (2007). Sensitive detection of FGFR3 mutations in bladder cancer and urine sediments by peptide nucleic acid-mediated real-time PCR clamping. Biochem Biophys Res Commun.

[17] Hyrup B, Nielsen PE (1996). Peptide nucleic acids (PNA): synthesis, properties and potential applications. Bioorg Med Chem.

[18] Maemondo M, Inoue A, Kobayashi K, Sugawara S, Oizumi S, Isobe H, Gemma A, Harada M, Yoshizawa H, Kinoshita I, Fujita Y, Okinaga S, Hirano H (2010). Gefitinib or chemotherapy for non-small-cell lung cancer with mutated EGFR. The New England journal of medicine.

[19] Nagai Y, Miyazawa H, T Huqun Tanaka, Udagawa K, Kato M, Fukuyama S, Yokote A, Kobayashi K, Kanazawa M, Hagiwara K (2005). Genetic heterogeneity of the epidermal growth factor receptor in non-small cell lung cancer cell lines revealed by a rapid and sensitive detection system, the peptide nucleic acid-locked nucleic acid PCR clamp. Cancer research.

[20] Thiede C, Bayerdorffer E, Blasczyk R, Wittig B, Neubauer A (1996). Simple and sensitive detection of mutations in the ras proto-oncogenes using PNA-mediated PCR clamping. Nucleic Acids Res.

[21] Dumonceau JM, Polkowski M, Larghi A, Vilmann P, Giovannini M, Frossard JL, Heresbach D, Pujol B, Fernandez-Esparrach G, Vazquez-Sequeiros E, Gines A (2011). European Society of Gastrointestinal E. Indications, results, and clinical impact of endoscopic ultrasound (EUS)-guided sampling in gastroenterology: European Society of Gastrointestinal Endoscopy (ESGE) Clinical Guideline. Endoscopy.

[22] Krishna NB, LaBundy JL, Saripalli S, Safdar R, Agarwal B (2009). Diagnostic value of EUS-FNA in patients suspected of having pancreatic cancer with a focal lesion on CT scan/MRI but without obstructive jaundice. Pancreas.

[23] Maluf-Filho F, Kumar A, Gerhardt R, Kubrusly M, Sakai P, Hondo F, Matuguma SE, Artifon E, Monteiro da Cunha JE, MC Cesar Machado, Ishioka S, Forero E (2007). Kras mutation analysis of fine needle aspirate under EUS guidance facilitates risk stratification of patients with pancreatic mass. Journal of clinical gastroenterology.

[24] Choi HJ, Moon JH, Kim HK, Lee YN, Lee TH, Cha SW, Cho YD, Park SH (2016.). KRAS mutation analysis by next-generation sequencing in endoscopic ultrasound-guided sampling for solid liver masses. Journal of gastroenterology and hepatology.

[25] Kobunai T, Watanabe T, Yamamoto Y, Eshima K (2010). The frequency of KRAS mutation detection in human colon carcinoma is influenced by the sensitivity of assay methodology: a comparison between direct sequencing and real-time PCR. Biochem Biophys Res Commun.

[26] Pellise M, Castells A, Gines A, Sole M, Mora J, Castellvi-Bel S, Rodriguez-Moranta F, Fernandez-Esparrach G, Llach J, Bordas JM, Navarro S, Pique JM (2003). Clinical usefulness of KRAS mutational analysis in the diagnosis of pancreatic adenocarcinoma by means of endosonography-guided fine-needle aspiration biopsy. Aliment Pharmacol Ther.

[27] Jiao Y, Shi C, Edil BH, de Wilde RF, Klimstra DS, Maitra A, Schulick RD, Tang LH, Wolfgang CL, Choti MA, Velculescu VE, Diaz LA, Vogelstein B, Kinzler KW, Hruban RH, Papadopoulos N (2011). DAXX/ATRX MEN1, and mTOR pathway genes are frequently altered in pancreatic neuroendocrine tumors. Science.

